# Probing the structural evolution of ruthenium doped germanium clusters: Photoelectron spectroscopy and density functional theory calculations

**DOI:** 10.1038/srep30116

**Published:** 2016-07-21

**Authors:** Yuanyuan Jin, Shengjie Lu, Andreas Hermann, Xiaoyu Kuang, Chuanzhao Zhang, Cheng Lu, Hongguang Xu, Weijun Zheng

**Affiliations:** 1 Department of Physics and Optoelectronic Engineering, Yangtze University, Jingzhou 434023, China; 2 Department of Physics, Nanyang Normal University, Nanyang 473061, China; 3 State Key Laboratory of Molecular Reaction Dynamics, Institute of Chemistry, Chinese Academy of Sciences, Beijing 100190, China; 4 Centre for Science at Extreme Conditions and SUPA, School of Physics and Astronomy, The University of Edinburgh, Edinburgh EH9 3JZ, United Kingdom; 5 Institute of Atomic and Molecular Physics, Sichuan University, Chengdu 610065, China; 6 Department of Physics and High Pressure Science and Engineering Center, University of Nevada, Las Vegas, Nevada 89154, United States

## Abstract

We present a combined experimental and theoretical study of ruthenium doped germanium clusters, RuGe_*n*_^−^ (*n* = 3–12), and their corresponding neutral species. Photoelectron spectra of RuGe_*n*_^−^ clusters are measured at 266 nm. The vertical detachment energies (VDEs) and adiabatic detachment energies (ADEs) are obtained. Unbiased CALYPSO structure searches confirm the low-lying structures of anionic and neutral ruthenium doped germanium clusters in the size range of 3 ≤ *n* ≤ 12. Subsequent geometry optimizations using density functional theory (DFT) at PW91/LANL2DZ level are carried out to determine the relative stability and electronic properties of ruthenium doped germanium clusters. It is found that most of the anionic and neutral clusters have very similar global features. Although the global minimum structures of the anionic and neutral clusters are different, their respective geometries are observed as the low-lying isomers in either case. In addition, for *n* > 8, the Ru atom in RuGe_*n*_^−/0^ clusters is absorbed endohedrally in the Ge cage. The theoretically predicted vertical and adiabatic detachment energies are in good agreement with the experimental measurements. The excellent agreement between DFT calculations and experiment enables a comprehensive evaluation of the geometrical and electronic structures of ruthenium doped germanium clusters.

Cluster is a new state of aggregation, which can be regarded as the intermediate phases between atoms or molecules and bulk solids^1^,^2^. A systematic study of clusters can provide valuable information on the evolution of the structural and electronic properties when isolated atoms or molecules become larger agglomerations, and bridge many fields of physics, such as atomic, molecular, and condensed-matter physics^3^. The central issue in cluster science is the determination of the true global minimum structures^4^. However, as the size of cluster increasing, finding the global minimum geometries becomes increasingly difficult due to the much increased complexity of the potential surface as well as the rapid increase of the number of low-lying isomers^5^,^6^. Luckily, the combined experimental and theoretical photoelectron spectroscopy approach has become a very effective method to identify the cluster structures, which has successfully determined the true global-minimum structures for various clusters ranging from small or medium-sized cluster to large sized cluster^7^,^8^,^9^,^10^,^11^,^12^.

Among the various classes of cluster materials, germanium-based clusters have attracted considerable experimental and theoretical attention because of that germanium is one of the most potential alternatives to silicon in microelectronic industry^13^,^14^. However, pure germanium clusters can not form stable fullerene-like cage structures and are unsuitable as a building block of self-assembly materials^15^,^16^. In order to generate and stabilize Ge cage structures, numerous experimental and theoretical investigations have been performed on transition metal (TM)-doped germanium clusters, similar to the case of TM-doped silicon clusters^11^,^12^,^17^,^18^,^19^,^20^,^21^,^22^,^23^,^24^,^25^. It is found that the TM-doped germanium clusters reveal different growth pattern from the TM-doped silicon clusters^19^,^26^. The critical size of TM-encapsulated Si_*n*_ structures is generally suggested to appear at *n* = 12^10^,^27^,^28^,^29^, while the TM-doped Ge_*n*_ clusters can form endohedral structures when *n *≤ 10^17^,^18^,^19^,^20^,^21^,^22^,^23^,^24^,^25^. Using solution chemistry methods, Wang *et al*.^17^ successfully synthesized the intermetalloid CoGe_10_^3−^ cluster with a *D*_*5h*_ pentagonal prism structure. Zhou *et al*.^18^ performed experimental measurements by using the standard Schlenk-line techniques and reported another pentagonal prismatic Zintl ion cage encapsulating an interstitial iron atom, FeGe_10_^3−^. Based on the anion photoelectron spectroscopy in combination with density functional theory (DFT) calculations, Deng *et al*.^11^ studied the structural, electronic and magnetic properties of VGe_*n*_^*−*/0^ (*n* = 3–12) clusters and suggested that the endohedral structures occur from *n* = 9 and a *D*_*3d*_ distorted hexagonal prism cage structure is formed at *n* = 12. They also observed that the critical size of the transition from exo- to endohedral structures is *n* = 9 for both anionic and neutral CoGe_*n*_ (*n* = 2–11) clusters^12^. Despite the much advances of 3*d* TM-doped germanium clusters, relatively little is known about the 4*d* TM-doped germanium clusters.

Very recently, Espinoza-Quintero *et al*.^30^ successfully synthesized the 12-vertex endohedral cluster RuGe_12_^3−^, a previously unknown 3-connected polyhedral geometry of *D*_*2d*_-symmetry, by the reaction of an ethylenediamine solution of K_4_Ge_9_ with [Ru(COD){*η*^3^-CH_3_C-(CH_2_)_2_}_2_] (COD = 1,5-cyclooctadiene). In order to confirm the geometries and electronic properties observed in their experimental measurements, Goicoechea and McGrady^31^ performed DFT calculations on MSi_12_ and MGe_12_ and concluded that the bicapped pentagonal prism structure of RuGe_12_^3−^ dominates the structural landscape for high valence electron counts (57–60). Nevertheless, up to now, there are no systematic investigations on neutral or single charged Ru-doped germanium clusters. There are still several essential open questions about the Ru-doped germanium clusters: How clusters grow with the increasing number of germanium atoms? What is the size of the smallest endohedral cage structures? What are the charge properties of the endohedral cage structures?

As an effort to address the above questions, here we report a combined photoelectron spectroscopy and DFT study on Ru-doped germanium clusters: RuGe_*n*_^−^ and RuGe_*n*_ (*n* = 3–12). The vertical detachment energy (VDE) and adiabatic detachment energy (ADE) of RuGe_*n*_^−^ are estimated from their photoelectron spectra. The structures of RuGe_*n*_^−^ and RuGe_*n*_ are assigned by the comparison of the theoretical simulations and experimental measurements. The thermodynamic stabilities of the obtained global minima are checked by analyzing the average binding energies (*E*_*b*_) and the second energy difference (∆^2^*E*). The natural population analysis is conducted to trace the negative charge dense regions in the neutral and anionic forms.

## Results and Discussion

### Experimental results

**Figure 1:**
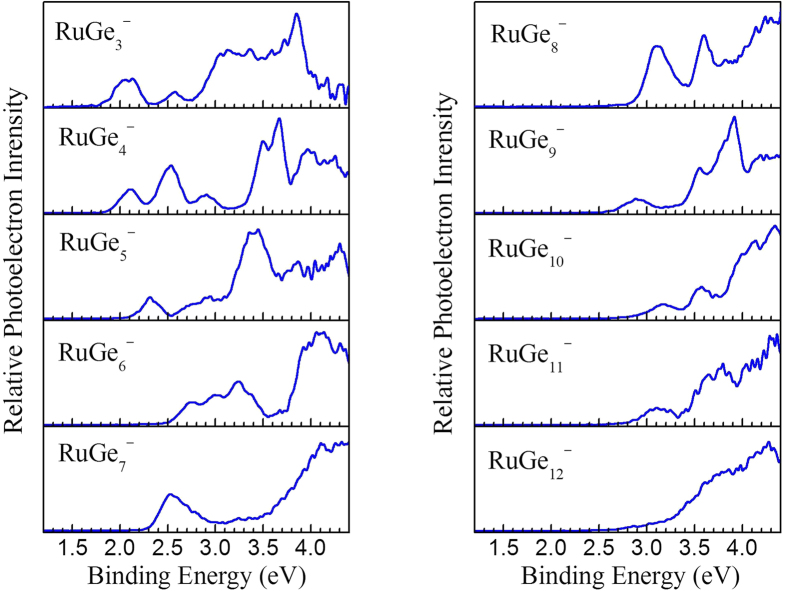
Experimental photoelectron spectra of RuGe_*n*_^−^ (*n* = 3–12) clusters recorded with 266 nm photons.

The photoelectron spectra of RuGe_*n*_^−^ clusters from *n* = 3 to 12 measured at 266 nm photons are presented in Fig. 1. In each spectrum, the first peak represents the transition from the ground electronic state of the cluster anion to that of the corresponding neutral species, and the other peaks with higher binding energy denote transitions to excited electronic states of the neutral clusters. The VDE is evaluated from the first peak. Meanwhile, the ADE of each cluster is estimated by adding the instrumental resolution to the binding energy which is the interaction of the binding energy axis and a straight line drawn along the leading edge of the first peak. The experimental VDE and ADE values of RuGe_*n*_^−^ clusters from photoelectron spectra in Fig. 1 are summarized in Table 1.

In the spectrum of RuGe_3_^−^, the first peak occurs at around 2.08 eV, which gives the VDE. After this, there is a weak peak at 2.57 eV and then a broadened area with several obscure peaks between 3.00 and 4.00 eV. From the photoelectron spectrum of RuGe_4_^−^, the VDE is about 2.12 eV. The second peak at 2.52 eV is stronger than the first one whereas the third weak peak occurs at 2.90 eV. The spectrum of RuGe_5_^−^ reveals that the VDE is about 2.32 eV and a second smaller peak locates at 2.95 eV. For RuGe_6_^−^, the VDE is about 2.75 eV and the front three peaks of the photoelectron spectrum are very close, followed by a broadened area between 3.90 and 4.40 eV. In the case of RuGe_7_^−^, the spectrum exhibits a major peak at 2.53 eV, which is the VDE. For RuGe_8_^−^, the VDE is about 3.10 eV followed by a sharp peak at 3.62 eV. The photoelectron spectrum of RuGe_9_^−^ reveals the first weak peak at about 2.89 eV and a second small peak at 3.54 eV. The spectrum of RuGe_10_^−^ is similar to that of RuGe_9_^−^, in with a VDE is 3.17 eV and a second peak at 3.58 eV. In the case of RuGe_11_^−^, there is a broad area between 2.80 and 3.30 eV and the VDE is about 3.14 eV. For RuGe_12_^−^, the intensity slowly increases from 2.60 to 3.81 eV and the VDE is around 3.81 eV.

### Theoretical results

**Figure 2:**
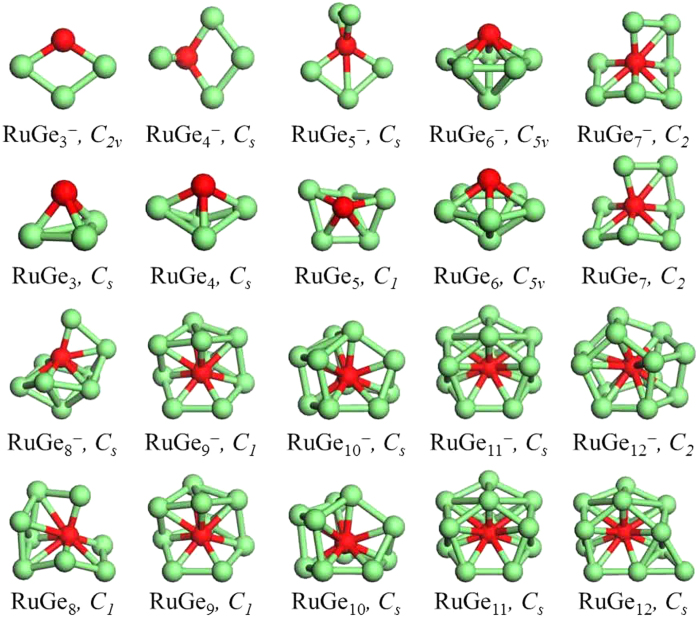
Global minimum structures of the RuGe_*n*_^−^ and RuGe_*n*_ (*n* = 3–12), along with the point group symmetries. The green balls are Ge atoms and the red balls are Ru atoms.

The optimized global minima of all RuGe_*n*_^−^ and RuGe_*n*_ (*n* = 3–12) clusters as obtained at the PW91/LANL2DZ level are plotted in Fig. 2. The geometries and relative stabilities of the low-lying isomers of each cluster are shown in Figs S1 and S2 in the Supporting Information (SI). The electronic states, point group symmetries and relative energies of all the considered clusters are summarized in Table S1 in the SI. The theoretical VDE and ADE values of the global minimum anions are listed in Table 1, in comparison with the experimental data. From Table 1, for all the ground state species, the computational VDE values are in good agreement with the experimental data, lending considerable credence to their structures.

As shown in Fig. 2, the global minimum structures of the neutral RuGe_*n*_ clusters are nearly identical to those of RuGe_*n*_^−^ when *n* = 6, 7, 9, 10, 11, while the structures of RuGe_*n*_ (*n* = 3–5, 9, 12) are different from their corresponding anions. However, both the anionic and neutral RuGe_*n*_^−/0^ clusters display the following structural trends: the global minimum RuGe_*n*_^−/0^ clusters with *n* = 3–6 have exohedral or Ru-capped geometries (except for RuGe_5_^−^); RuGe_7_^−/0^ and RuGe_8_^−/0^ possess half-encapsulated structures; and endohedral geometries are adopted for the larger clusters with *n* = 9–12. In particular, the global minimum structure of RuGe_10_^−/0^ is a 3-connected *C*_*s*_-symmetric polyhedral cage encapsulating the Ru atom in the center. As well as, a *C*_*2*_-symmetric ground state RuGe_12_^−^ also reveals a 3-connected polyhedral geometry, which mirrors the structure of trianionic RuGe_12_^3−^ reported by Espinoza-Quintero *et al*.^30^. Unfortunately, except for this, there are no more available experimental data to compare with our calculations for RuGe_*n*_^−/0^.

### Comparison between experiment and theory

**Figure 3:**
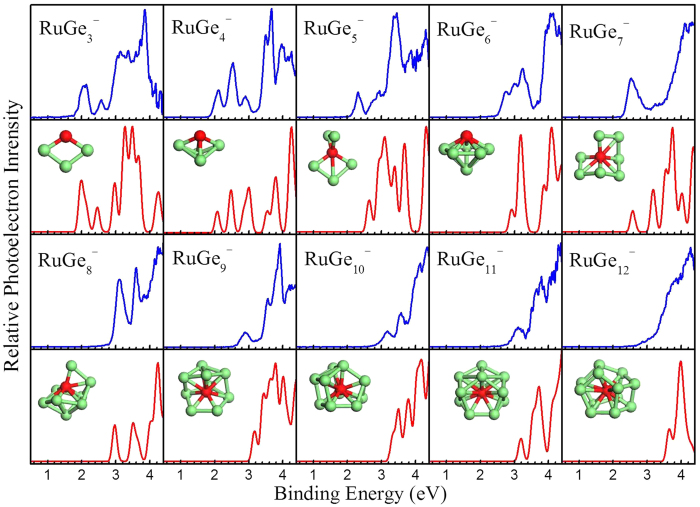
Simulated (red color) photoelectron spectra for RuGe_*n*_^−^ (*n* = 3–12) clusters, along with the experimental spectra (blue color) from Fig. 1 for comparison. A uniform Gaussian broadening of 0.15 eV is chosen for all the simulated spectra.

In order to prove the credibility of the obtained ground state structures, the photoelectron spectra of RuGe_*n*_^−^ (*n* = 3–12) are simulated and displayed in Fig. 3, along with the experiment spectra from Fig. 1 for comparison. In general, the simulated spectra are in overall satisfying agreement with the measured photoelectron spectra, which certify the validity of the present theoretical results.

For RuGe_3_^−^, the first two discrete peaks of the simulated spectrum are red-shifted compared to the experimental results, however, the others agree well. In the simulated spectrum of RuGe_4_^−^, the first five peaks are all in excellent agreement with the experimental measurements. For RuGe_5_^−^, the first peak is located at 2.64 eV, a little higher than the experimental value. The following peak is very broad, in accordance with experimental determination, and originates from several individual excitations. In the case of RuGe_6_^−^, the essential features of the experimental spectrum are well reproduced by DFT simulation. In the calculated photoelectron spectrum of RuGe_7_^−^, the first peak is in excellent agreement with the experimental measurement, while the others are somewhat different. In the theoretical spectrum of RuGe_8_^−^, three major peaks are obtained, which are in very good agreement with experiment. In the case of RuGe_9_^−^, the overall experimental spectrum is well reproduced by theoretical calculations; however, the first calculated peak at 3.17 eV is a little higher in energy and thus less distinct than the first experimental peak. In the theoretical spectrum of RuGe_10_^−^, three obvious peaks can be clearly seen, in accordance with the experimental data. For RuGe_11_^−^, the DFT calculation successfully reproduces the experimental trend and yields two obvious peaks. The simulated spectrum of RuGe_12_^−^ has an onset very similar to the experimental spectrum, and reproduces a small shoulder before the main peak.

**Figure 4:**
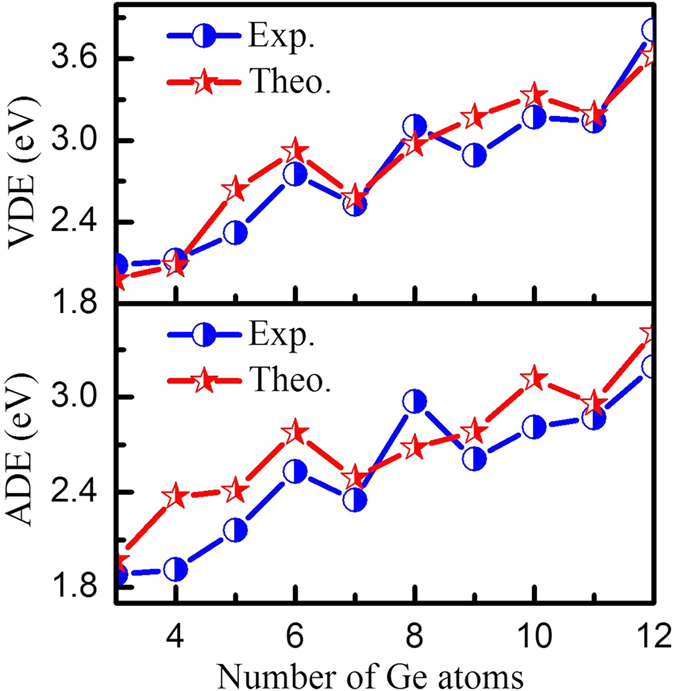
Vertical detachment energies (VDEs) and adiabatic detachment energies (ADEs) of RuGe_*n*_^−/0^ (*n* = 3–12) clusters: blue circles, experiment; red pentacles, theory.

For the sake of a clearer comparison to experiment, the experimental and computed VDEs and ADEs as functions of cluster size are shown in Fig. 4. In the experimental VDE curve, an overall increase can be seen from 1.98 to 3.62 eV across the different clusters, but features local minima at RuGe_7_^−^, RuGe_9_^−^, and RuGe_11_^−^. The theoretical VDEs reproduce this trend generally well, bar for the local minimum at RuGe_9_^−^. In general, the ADE value of an anionic cluster is equal to the adiabatic electron affinity (AEA) of the corresponding neutral species when their geometries are similar to each other. From experiment, it can be seen that ADE values keep rising from 1.80 to 2.45 eV for RuGe_*n*_^*−*^ (*n* = 3–6); thereafter this trend slows down, with an increase from 2.53 to 3.11 eV as the number of germanium atoms increases from 9 to 12. The ADE shows oscillatory from *n* = 6 to 9, with local minimum at RuGe_7_^−^ (2.27 ± 0.08 eV) and RuGe_9_^−^ (2.53 ± 0.08 eV). Again, the experimental trend is well reproduced by DFT simulations, but with an overestimation of about 0.46 eV at RuGe_4_^−^ and 0.30 eV at RuGe_10_^−^ respectively. From electronic points of view, RuGe_7_^−^ and RuGe_9_^−^ stand out as unique species with appreciable stability. For a given neutral cluster, lower AEA corresponds to higher stability. This indicates, in other words, that the neutral RuGe_7_ and RuGe_9_ clusters are stable.

### Stabilities and electronic properties

**Figure 5:**
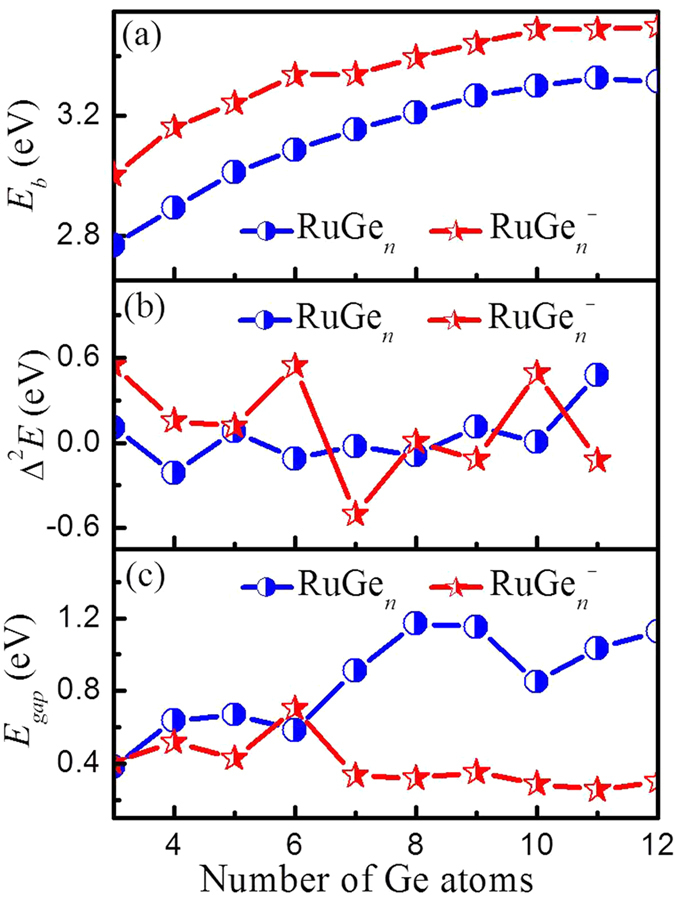
Size dependences of average binding energies (*E*_*b*_), second order difference (Δ^2^*E*) and HOMO–LUMO gaps (*E*_*gap*_) for the global minimum RuGe_*n*_^−/0^ (*n* = 3–12) clusters.

The thermodynamic stabilities of the RuGe_*n*_^−^ and RuGe_*n*_ (*n* = 3–12) clusters can be explored by investigating two thermodynamic parameters, namely the average binding energy and the second energy difference. The *E*_*b*_ and ∆^2^*E* of RuGe_*n*_^−/0^ clusters are defined as follows:


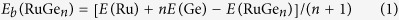



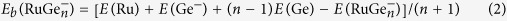



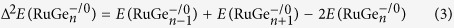


where *E* is the energy of the corresponding atom or cluster. The behaviors of calculated *E*_*b*_ and ∆^2^*E* values as function of cluster size for RuGe_*n*_^−^ and RuGe_*n*_ clusters are shown in Fig. 5(a,b), respectively. From Fig. 5(a), the binding energy per atom increases monotonously, but saturates for *n* > 9, with increasing number of Ge atoms, suggesting that the formation of larger clusters is favorable over smaller clusters. For all cluster sizes, *E*_*b*_ values of anionic clusters are higher than those of the neutral species. This implies that as soon as a neutral cluster gains an extra electron, it becomes more stable. From Fig. 5(b), one can see stronger oscillations of the second energy difference in the anionic than the neutral clusters. An obvious odd−even oscillation for anions is found from *n* = 5 to 11. The peaks for RuGe_6_^−^, RuGe_8_^−^ and RuGe_10_^−^ indicate that these are more stable than their neighboring sized clusters.

The energy gap (*E*_*gap*_) between the highest occupied molecular orbital (HOMO) and the lowest unoccupied molecular orbital (LUMO) indicates the ability of electrons to jump from occupied orbitals to unoccupied orbitals. As shown in Table S1 and Fig. 5(c), the HOMO–LUMO gap values of RuGe_*n*_^−/0^ clusters range between 0.26 and 1.17 eV. Regarding cluster size, the *E*_*gap*_ values of neutral species with *n* > 6 are much larger than those of the small clusters. In contrast, the *E*_*gap*_ values of the larger sized anions are smaller than those of the small species. It stands out that neutral RuGe_8_, RuGe_9_ and RuGe_12_ have the largest HOMO–LUMO gaps, implying that they are (relatively) more stable based on their electronic structure.

**Figure 6:**
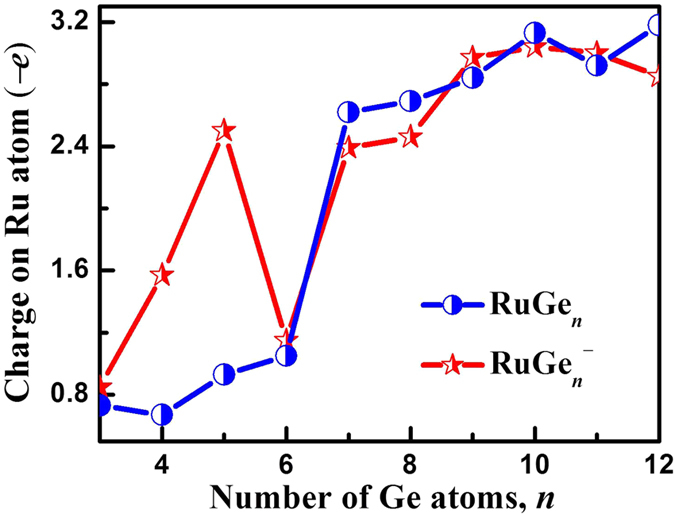
Natural charge populations of the Ru atom for the global minimum structures of RuGe_*n*_^−/0^ (*n* = 3–12) clusters.

In order to examine the charge transfers, we next conduct the natural population analyses for the most stable isomers of the anionic and neutral RuGe_*n*_^−/0^ (*n* = 3–12) clusters. The results are summarized in Fig. 6. For the anionic clusters, it is found that, for RuGe_3_^−^, the negative charge is localized on both Ru atom and the Ge_*n*_ framework. For the larger anions, there is increasing electron transfer from the Ge_*n*_ framework to the Ru atom. For cluster size of *n* = 7–12, in particular, the negative charge on the Ru atom increases significantly, suggesting that there is substantial electron transfer from the Ge_*n*_ framework to the Ru atom. In the case of neutral clusters, the electron transfer from the Ge_*n*_ atoms to the Ru atom occurs for all cluster size and the charges on Ru are much larger for clusters with *n* = 7–12. The electron transfer from the Ge_*n*_ framework to the Ru atom is related to the formation of endohedral structures.

## Conclusions

In summary, we have reported a systematic study of the relative stability and electronic properties of ruthenium doped germanium clusters in the size range of 3 ≤ *n* ≤ 12. Photoelectron spectra of anionic RuGe_*n*_^−^ clusters are measured at 266 nm. Unbiased structure searches reveal quite similar global minimum structures for both anionic and neutral clusters. Excellent agreement between theoretical calculations and experimental measurements is found. The global minimum anionic RuGe_*n*_^−^ clusters with *n* = 3–6 as well as their neutral counterparts have Ru-capped structures (except for RuGe_5_^−^); half-encapsulated structures are found for RuGe_7_^−/0^ and RuGe_8_^−/0^; and the larger clusters RuGe_*n*_^−/0^ (*n* = 9–12) feature endohedral geometries. From natural population analyses we see that, for the larger clusters with *n* = 7–12, the negative charge on the Ru atom increases significantly, suggesting that there is more electron transfer from the Ge_*n*_ framework to the Ru atom, which stabilizes the formation of endohedral cage.

### Experimental and computational methods

Experiments are carried out on a home-built instrument consisting of a laser vaporization cluster source, a time-of-flight (TOF) mass spectrometer, and a magnetic-bottle photoelectron spectrometer, which have been described elsewhere^32^. In the laser vaporization source, the anionic Ru−Ge clusters are produced by laser ablation of a rotating translating disk target (13 mm diameter, Ru/Ge mole ratio 1:2) with a nanosecond Nd:YAG laser (Continuum Surelite II-10). To cool the generated cluster, helium gas with ~4 atm backing pressure is allowed to expand through a pulsed valve (General Valve Series 9) into the source. At the TOF mass spectrometer, the thus-formed cluster anions are mass-analyzed. The RuGe_*n*_^−^ (*n* = 3–12) clusters are individually selected with a mass gate, decelerated by a momentum decelerator, and crossed with the beam of a Nd:YAG laser (Continuum Surelite II-10, 266 nm) in the photodetachment setup. The magnetic-bottle photoelectron spectrometer is used to energy-analyze the electrons from the photodetachment. The resolution of the magnetic-bottle photoelectron spectrometer is about 40 meV at electron kinetic energy of 1 eV.

Theoretically, the structure searches for anionic RuGe_*n*_^−^ (*n* = 3–12) clusters and their neutral states are performed using CALYPSO method^33^,^34^,^35^,^36^,^37^. This method is based on globally minimizing potential energy surfaces, merging *ab initio* total energy calculations with CALYPSO cluster prediction through particle swarm optimization. It has been successful in correctly predicting structures for various systems^35^,^36^,^38^. The low-lying isomers of RuGe_*n*_^−/0^ (*n* = 3–12) found in the searches are further optimized using DFT with the PW91 functional^39^. The LANL2DZ basis set is used for both Ru and Ge atoms. Spin multiplicities (up to septet and octet) are considered for refined structure optimization. Vibrational frequency calculations are used to verify the nature of real local minima. To further evaluate the relative energies of the low-lying structures, single-point calculations were carried out by employing the larger def2-TZVP basis set. Excitation energies of the neutral cluster are calculated using the time-dependent DFT (TDDFT) method at the corresponding anionic structure^40^. All calculations are carried out with the Gaussian 09 package^41^. The PW91/LANL2DZ theory show superior results in terms of structures and binding energies for the species considered here and are therefore used for direct comparison with the experiment.

## Additional Information

**How to cite this article**: Jin, Y. *et al*. Probing the structural evolution of ruthenium doped germanium clusters: Photoelectron spectroscopy and density functional theory calculations. *Sci. Rep.* **6**, 30116; doi: 10.1038/srep30116 (2016).

## Supplementary Material

Supplementary Information

## Figures and Tables

**Table 1:** Vertical detachment energies (VDEs) and adiabatic detachment energies (ADEs) of the ground state RuGe_*n*_^−^ (*n* = 3–12) clusters estimated from their photoelectron spectra.

Cluster	VDE (eV)		ADE (eV)	
	Exp.	Theo.	Exp.	Theo.
RuGe_3_^−^	2.08 ± 0.08	1.98	1.80 ± 0.08	1.97
RuGe_4_^−^	2.12 ± 0.08	2.08	1.83 ± 0.08	2.37
RuGe_5_^−^	2.32 ± 0.08	2.64	2.08 ± 0.08	2.41
RuGe_6_^−^	2.75 ± 0.08	2.64	2.45 ± 0.08	2.77
RuGe_7_^−^	2.53 ± 0.08	2.58	2.27 ± 0.08	2.49
RuGe_8_^−^	3.10 ± 0.08	2.97	2.89 ± 0.08	2.68
RuGe_9_^−^	2.89 ± 0.08	3.17	2.53 ± 0.08	2.78
RuGe_10_^−^	3.17 ± 0.08	3.33	2.73 ± 0.08	3.11
RuGe_11_^−^	3.14 ± 0.08	3.19	2.79 ± 0.08	2.96
RuGe_12_^−^	3.81 ± 0.08	3.62	3.11 ± 0.08	3.41
