# Supplementary material for: Probing the structural evolution of ruthenium doped germanium clusters: Photoelectron spectroscopy and density functional theory calculations
Source: Sci Rep. 2016 Jul 21;6:30116. doi: 10.1038/srep30116 (PMC4954966; doi:10.1038/srep30116)
Supplement: Supplementary Information [file srep30116-s1.doc]

**Supporting Information**

**Probing the structural evolution of** **ruthenium doped germanium clusters: Photoelectron spectroscopy and** **density functional theory** **calculations**

Yuanyuan Jin,^1,2^ Shengjie Lu,^3^ Andreas Hermann,^4^ Xiaoyu Kuang,^5^,* Chuanzhao Zhang,^1^ Cheng Lu,^2,6^,* Hongguang Xu,^3^,* and Weijun Zheng^3^

^1^ Department of Physics and Optoelectronic Engineering, Yangtze University, Jingzhou 434023, China

^2^Department of Physics, Nanyang Normal University, Nanyang 473061, China

^3^State Key Laboratory of Molecular Reaction Dynamics, Institute of Chemistry, Chinese Academy of Sciences, Beijing 100190, China

^4^Centre for Science at Extreme Conditions and SUPA, School of Physics and Astronomy, The University of Edinburgh, Edinburgh EH9 3JZ, United Kingdom

^5^Institute of Atomic and Molecular Physics, Sichuan University, Chengdu 610065, China

^6^Department of Physics and High Pressure Science and Engineering Center, University of Nevada, Las Vegas, Nevada 89154, United States

*Correspondence author. E-mail: [scu_kuang@163.com](mailto:scu_kuang@163.com) (Xiaoyu Kuang), [lucheng@calypso.cn](mailto:lucheng@calypso.cn) (Cheng Lu) and xuhong@iccas.ac.cn (Hongguang Xu)


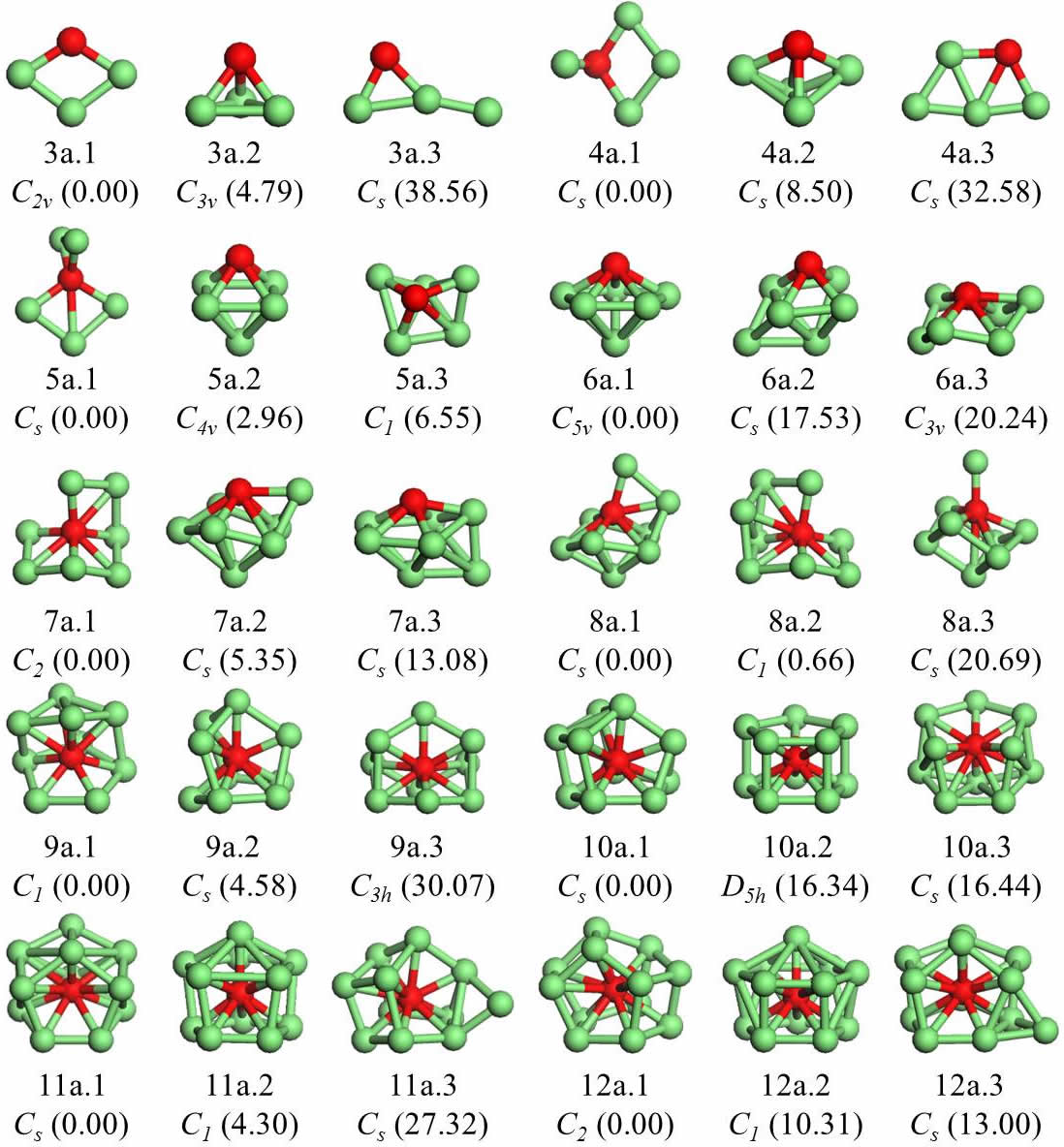


**Figure S1.** Optimized structures of the anionic RuGe*_n_*^−^ (*n* = 3−12), along with the point group symmetries and relative energies (kcal/mol). Relative energies are given at PW91/LANL2DZ level of theory. The green balls are Ge atoms and the red balls are Ru atoms.


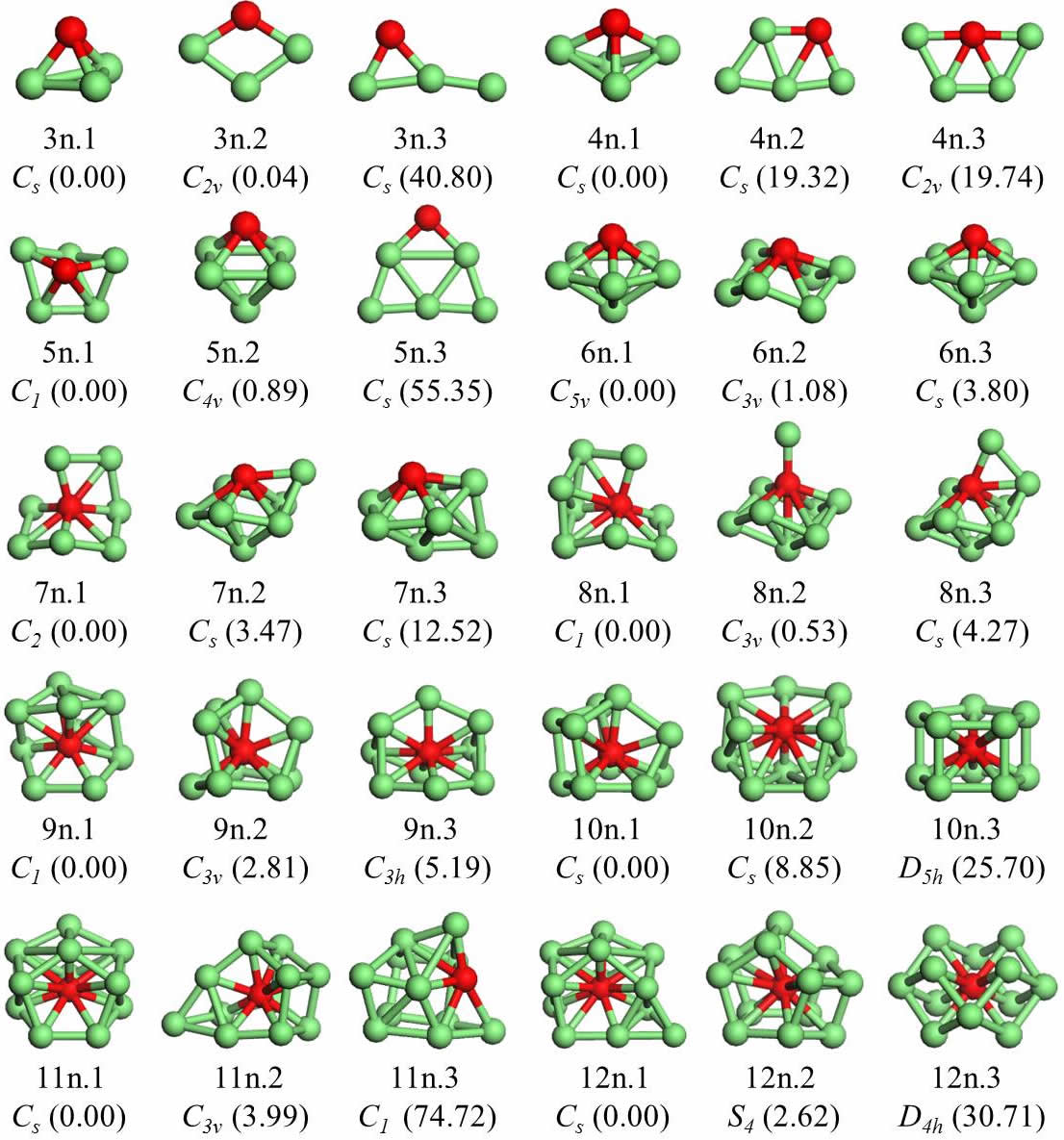


**Figure S2.** Optimized structures of the neutral RuGe*_n_* (*n* = 3−12), along with the point group symmetries and relative energies (kcal/mol). Relative energies are given at PW91/LANL2DZ level of theory. The green balls are Ge atoms and the red balls are Ru atoms.

| RuGe*_n_*^−^ | | | | | | RuGe*_n_* | | | | | | |
| --- | --- | --- | --- | --- | --- | --- | --- | --- | --- | --- | --- | --- |
| Iso. | State | Sym. | Δ*E*  (kcal/mol) | *E_gap_*  (eV) | *Q*  (*e*) | Iso. | State | Sym. | Δ*E*  (kcal/mol) | *E_gap_*  (eV) | *Q*  (*e*) |  |
| **3a.1** | **^2^B_1_** | ***C_2v_*** | **0.00** | **0.41** | **-0.85** | **3n.1** | **^3^A*"*** | ***C_s_*** | **0.00** | **0.38** | **-0.73** |  |
| 3a.2 | ^4^A_1_ | *C_3v_* | 4.79 |  |  | 3n.2 | ^3^B**_1_** | *C_2v_* | 0.04 |  |  |  |
| 3a.3 | ^4^A*"* | *C_s_* | 38.56 |  |  | 3n.3 | ^5^A*"* | *C_s_* | 40.80 |  |  |  |
| **4a.1** | ^2^A*'* | ***C_s_*** | **0.00** | **0.52** | **-1.57** | **4n.1** | **^3^A*"*** | ***C_s_*** | **0.00** | **0.64** | **-0.67** |  |
| 4a.2 | ^4^A*"* | *C_s_* | 8.50 |  |  | 4n.2 | ^3^A' | *C_s_* | 19.32 |  |  |  |
| 4a.3 | ^4^A*"* | *C_s_* | 32.58 |  |  | 4n.3 | ^3^A_2_ | *C_2v_* | 19.74 |  |  |  |
| **5a.1** | **^2^A*"*** | ***C_s_*** | **0.00** | **0.43** | **-2.50** | **5n.1** | **^1^A** | ***C_1_*** | **0.00** | **0.67** | **-0.93** |  |
| 5a.2 | ^2^B_1_ | *C_4v_* | 2.96 |  |  | 5n.2 | ^3^B**_1_** | *C_4v_* | 0.89 |  |  |  |
| 5a.3 | ^2^A | *C_1_* | 6.55 |  |  | 5n.3 | ^5^A*"* | *C_s_* | 55.35 |  |  |  |
| **6a.1** | **^2^A_1_** | ***C_5v_*** | **0.00** | **0.70** | **-1.15** | **6n.1** | **^1^A_1_** | ***C_5v_*** | **0.00** | **0.59** | **-1.05** |  |
| 6a.2 | ^2^A*'* | *C_s_* | 17.53 |  |  | 6n.2 | ^1^A**_1_** | *C_3v_* | 1.08 |  |  |  |
| 6a.3 | ^4^A_1_ | *C_3v_* | 20.24 |  |  | 6n.3 | ^3^A*'* | *C_s_* | 3.80 |  |  |  |
| **7a.1** | **^2^B** | ***C_2_*** | **0.00** | **0.33** | **-2.39** | **7n.1** | **^1^A** | ***C_2_*** | **0.00** | **0.91** | **-2.62** |  |
| 7a.2 | ^2^A*'* | *C_s_* | 5.35 |  |  | 7n.2 | ^3^A*'* | *C_s_* | 3.47 |  |  |  |
| 7a.3 | ^2^A*'* | *C_s_* | 13.08 |  |  | 7n.3 | ^1^A*'* | *C_s_* | 12.52 |  |  |  |
| **8a.1** | **^2^A*'*** | ***C_s_*** | **0.00** | **0.32** | **-2.46** | **8n.1** | **^1^A** | ***C_1_*** | **0.00** | **1.17** | **-2.69** |  |
| 8a.2 | ^2^A | *C_1_* | 0.66 |  |  | 8n.2 | ^1^A**_1_** | *C_3v_* | 0.53 |  |  |  |
| 8a.3 | ^4^A*"* | *C_s_* | 20.69 |  |  | 8n.3 | ^1^A*'* | *C_s_* | 4.27 |  |  |  |
| **9a.1** | **^2^A** | ***C_1_*** | **0.00** | **0.35** | **-2.97** | **9n.1** | **^1^A** | ***C_1_*** | **0.00** | **1.15** | **-2.84** |  |
| 9a.2 | ^2^A*'* | *C_s_* | 4.58 |  |  | 9n.2 | ^1^A**_1_** | *C_3v_* | 2.81 |  |  |  |
| 9a.3 | ^4^A*'* | *C_3h_* | 30.07 |  |  | 9n.3 | ^1^A*'* | *C_3h_* | 5.19 |  |  |  |
| **10a.1** | **^2^A*'*** | ***C_s_*** | **0.00** | **0.29** | **-3.04** | **10n.1** | **^1^A*'*** | ***C_s_*** | **0.00** | **0.85** | **-3.13** |  |
| 10a.2 | ^4^A_1_*'* | *D_5h_* | 16.34 |  |  | 10n.2 | ^1^A*'* | *C_s_* | 8.85 |  |  |  |
| 10a.3 | ^2^A*'* | *C_s_* | 16.44 |  |  | 10n.3 | ^5^A_2_*"* | *D_5h_* | 25.70 |  |  |  |
| **11a.1** | **^2^A*'*** | ***C_s_*** | **0.00** | **0.26** | **-3.00** | **11n.1** | **^1^A*'*** | ***C_s_*** | **0.00** | **1.04** | **-2.92** |  |
| 11a.2 | ^2^A | *C_1_* | 4.30 |  |  | 11n.2 | ^1^A**_1_** | *C_3v_* | 3.99 |  |  |  |
| 11a.3 | ^4^A*"* | *C_s_* | 27.32 |  |  | 11n.3 | ^1^A | *C_1_* | 74.72 |  |  |  |
| **12a.1** | **^2^B** | ***C_2_*** | **0.00** | **0.30** | **-2.85** | **12n.1** | **^1^A*'*** | ***C_s_*** | **0.00** | **1.13** | **-3.18** |  |
| 12a.2 | ^2^A | *C_1_* | 10.31 |  |  | 12n.2 | ^3^A | *S_4_* | 2.62 |  |  |  |
| 12a.3 | ^2^A*"* | *C_s_* | 13.00 |  |  | 12n.3 | ^1^A**_1_**_g_ | *D_4h_* | 30.71 |  |  |  |

**Table S1.** Electronic states (State), point group symmetries (Sym.), relative energies (Δ*E*, kcal/mol), HOMO−LUMO gaps (*E_gap_*, eV), and charges on Ru atoms (*Q*, *e*) of the low-lying RuGe*_n_*^−^ and RuGe*_n_* (*n* = 3−12) isomers at PW91/LANL2DZ.
